# Quinidine, but Not Eicosanoid Antagonists or Dexamethasone, Protect the Gut from Platelet Activating Factor-Induced Vasoconstriction, Edema and Paralysis

**DOI:** 10.1371/journal.pone.0120802

**Published:** 2015-03-20

**Authors:** Ingmar Lautenschläger, Inéz Frerichs, Heike Dombrowsky, Jürgen Sarau, Torsten Goldmann, Karina Zitta, Martin Albrecht, Norbert Weiler, Stefan Uhlig

**Affiliations:** 1 Department of Anesthesiology and Intensive Care Medicine, University Medical Center Schleswig-Holstein, Campus Kiel, Kiel, Germany; 2 Division of Barrier Integrity, Research Center Borstel, Leibniz-Center for Medicine and Biosciences, Borstel, Germany; 3 Division of Mucosal Immunology and Diagnostic, Research Center Borstel, Leibniz-Center for Medicine and Biosciences, Borstel, Germany; 4 Division of Clinical and Experimental Pathology, Research Center Borstel, Leibniz-Center for Medicine and Biosciences, Borstel, Germany; 5 Institute of Pharmacology and Toxicology, Medical Faculty, RWTH Aachen University, Aachen, Germany; National Institute of Agronomic Research, FRANCE

## Abstract

Intestinal circulatory disturbances, atony, edema and swelling are of great clinical relevance, but the related mechanisms and possible therapeutic options are poorly characterized, in part because of the difficulties to comprehensively analyze these conditions. To overcome these limitations we have developed a model of the isolated perfused rat small intestine where all of these symptoms can be studied simultaneously. Here we used this model to study the role of eicosanoids, steroids and quinidine in platelet-activating factor (PAF)-induced intestinal disorders. A vascular bolus of PAF (0.5 nmol) triggered release of thromboxane and peptidoleukotrienes into the vascular bed (peak concentration 35 nM and 0.8 nM) and reproduced all symptoms of intestinal failure: mesenteric vasoconstriction, translocation of fluid and macromolecules from the vasculature to the lumen and lymphatics, intestinal edema formation, loss of intestinal peristalsis and decreased galactose uptake. All effects of PAF were abolished by the PAF-receptor antagonist ABT491 (2.5 μM). The COX and LOX inhibitors ASA and AA861 (500 μM, 10 μM) did not exhibit barrier-protective effects and the eicosanoid antagonists SQ29548 and MK571 (10 μM, each) only moderately attenuated the loss of vascular fluid, the redistribution to the lumen and the transfer of FITC dextran to the lumen. The steroid dexamethasone (10 μM) showed no barrier-protective properties and failed to prevent edema formation. Quinidine (100 μM) inhibited the increase in arterial pressure, stabilized all the intestinal barriers, and reduced lymph production and the transfer of FITC dextran to the lymph. While quinidine by itself reduced peristalsis, it also obviated paralysis, preserved intestinal functions and prevented edema formation. We conclude that quinidine exerts multiple protective effects against vasoconstriction, edema formation and paralysis in the intestine. The therapeutic use of quinidine for intestinal ailments deserves further study.

## Introduction

Intestinal failure as a consequence of endothelial and epithelial barrier dysfunction is a continuing problem in SIRS (systemic inflammatory response syndrome) and sepsis. No causal therapies exist for the treatment of intestinal edema and associated complications like disturbances in gut motility and enteral feeding, anastomotic leakage and translocation of pathogens. It is generally accepted that these pathophysiological alterations are caused by inflammatory mediators such as TNF-α, VEGF, thrombin, histamine, bradykinin, or the lipid mediator PAF. While some of them such as histamine and bradykinin can increase mesenteric microvascular permability and cause edema formation [1,2], only PAF has the ability to also cause gastrointestinal stasis [3,4], vasoconstriction [3,5,6], and vasocongestion [3,7,8]. Consequently, PAF is involved in the pathogenesis of many inflammatory bowel diseases such as neonatal necrotizing enterocolitis [9], ischemia-reperfusion injury [10], antibody-induced intestinal dysfunction [11] and sepsis [12]. However, little is known about the mechanisms responsible for these PAF-induced dysfunctions and causal therapeutic strategies that take into account the multiple dysfunctions caused by PAF have not been developed yet. The lack of studies in this area is exemplified by the fact that until now it is unknown whether anti-leukotriene strategies, steroids or other anti-inflammatory drugs can mitigate the PAF-induced vasoconstriction, edema formation and paralysis in the intestine.

One problem in studying the effects of PAF is that this mediator also activates leukocytes making it very difficult *in vivo* to distinguish between direct and indirect effects of PAF. In addition, it is technically demanding to follow mesenteric vasoconstriction, gastrointestinal edema and (paralytic) ileus *in vivo*. To overcome all these problems, we have recently established an isolated blood-free perfused rat small intestine model [3].

Since eicosanoids have been implicated in the development of intestinal damage [13–15], we first examined whether PAF liberates eicosanoids into the mesenteric perfusate. We next investigated the relevance of the eicosanoid pathway for the PAF-induced intestinal dysfunctions by using either cyclooxygenase (COX) and lipoxygenase (LOX) inhibitors or alternatively thromboxane receptor (TX) and leukotriene (LT) antagonists. We applied COX/LOX inhibitors or TX/LT antagonists in combination since cyclooxygenase inhibition may increase leukotriene metabolites [16,17], and frequently thromboxane and leukotrienes operate in concert.

In addition to eicosanoid inhibition, we studied two broadly effective anti-inflammatory drugs, i.e. dexamethasone and quinidine. Both of these drugs are known to attenuate PAF responses in the lungs [18,19] and there is some evidence that the steroid dexamethasone may exert beneficial effects in the intestine [20–22]. Even though quinolines may provoke multiple side effects [23], they are clinically used as antirheumatic (hydroxychloroquine), antimalarial (chloroquine) and antiarrhytmic (quinidine) drugs [24,25]. Notably, the quinolines quinine, quinidine and chloroquine exert potent anti-inflammatory effects in many organs such as lungs [18,26,27] and liver [28]. Therefore, we checked if these two broad anti-inflammatory agents are able to protect the gut from the PAF-induced intestinal failure. Remarkably, only quinidine, but not dexamethasone was effective against the multiple PAF-induced dysfunctions in the intestine: vasoconstriction, edema formation and fluid shifts, vascular leakage based on the distribution of FITC dextran, paralysis, and further physiological, biochemical and histological abnormalities.

## Materials and Methods

### Animals

Wistar rats were obtained from Charles River (Charles River Laboratories, Sulzfeld, Germany) and used as donors. For practical reasons we used female rats, exclusively. Multiple experiments, including those performed during the development of our model [3], have not indicated any effects of the menstrual cycle on the actions of PAF. The intestinal effects of PAF were reproducible at all times and occurred within minutes after the administration of this inflammatory mediator. For technical reasons (preparation procedure and insertion of cannulae into the small blood vessels and intestine) we used 10 to 12 weeks old rats weighing 223 ± 17g (mean ± SD). We did not use older rats, because lactose digestion and resorption of galactose decreases with aging in this species [29]. The study was conducted in agreement with the ethical requirements of the Animal Care Committee of the Ministry of Energy, Agriculture, the Environment and Rural Areas of Schleswig-Holstein, Germany and in direct accordance with the German Animal Protection Law. The protocols were approved by the Ministry of Energy, Agriculture, the Environment and Rural Areas of Schleswig-Holstein, Germany (Protocol: V312–72241.123–3(A47 and A56)). All efforts were made to minimize suffering. The animals were anesthetized by inhalation of sevoflurane and supplemented with an intramuscular injection of ketamine. Subsequent to the dissection of the intestine, a lethal dose of pentobarbital (100mg) was used for euthanasia.

### Study design

PAF is one of the few inflammatory mediators that can cause capillary leak and edema formation within minutes. In many cases, the effects of PAF are mediated by other lipid mediators such as ceramide or eicosanoids and they are prevented by dexamethasone and by quinolines [30]. Therefore, as summarized in the S1 Fig., we investigated the role of the eicosanoids thromboxane and leukotriene in PAF-induced intestinal failure as well as the potential protective effects of dexamethasone and quinidine. We used the model of the isolated perfused rat small intestine to perform three different series of experiments. In the first series, the liberation of eicosanoids from the intestine to the vascular compartment was quantified by enzyme linked immunosorbent assay (ELISA) in PAF-stimulated intestines (PAF, 0.5 nmol; group: ELISA-PAF) and in controls (group: ELISA-SOL). In the second series, we analyzed the PAF-induced changes in intestinal physiology and intestinal function (PAF, 0.5 nmol; group: PAF vs. ABT 491, 2.5 μM; group: PAF-RA) and the potential protective effects of COX and LOX inhibition (ASA, 500 μM / AA 861, 10 μM; group: COX/LOX-), thromboxane and leukotriene antagonism (SQ 29548, 10 μM / MK 571, 10 μM; group: TX/LT-), dexamethasone (Dexamethasone, 10 μM; group: DEXA) and quinidine (Quinidine, 100 μM; group: QD). All pretreatment compounds were administrated 20 minutes before stimulation with PAF. In the third series, experiments were stopped three minutes after stimulation with PAF alone (PAF, 0.5 nmol; group: PAF-63), or with dexamethasone (Dexamethasone, 10 μM; group: DEXA-63) or quinidine (Quinidine, 100 μM; group: QD-63) pretreatment, or at the equivalent time point without any challenge or pretreatment (group: SOL-63) to analyze PAF-induced edema formation and to evaluate the protective effect of dexamethasone and quinidine. In all groups, except for controls, PAF was administered 60 minutes after the perfusion initiation, i.e. after the equilibration of the perfusion system. PAF was applied as a bolus of 0.5 nmol via the mesenteric artery within 20 seconds. The experimental protocol is presented schematically in S2 Fig. and the detailed description of the groups is shown in S1 Table.

### Isolated perfusion and analysis

The small intestines of non-fasted anesthetized rats were isolated and perfused both vascularly (7.5 ml·min^-1^) and luminally (0.15 ml·min^-1^) in a specialized perfusion chamber (Hugo-Sachs Elektronik-Harvard Apparatus, Holliston, MA, USA) as described [3]. The principle of the perfusion system is shown in the S3 Fig. This set-up was designed to allow the assessment of fluid shifts and the macromolecular transfer of FITC-labeled dextran (150 kDa) within the vascular, lymphatic and luminal compartments. The pressures in the vascular and the luminal compartments were recorded continuously, while the lymphatic fluid drained from the lymphatic vessels without additional resistance. The vascular fluid losses to the lymphatics and the lumen within 10 minutes after stimulation were calculated from the cumulative weight measurements of the drained fluid. The transfer of vascular dextran as a measure of permeability and the resorption of galactose derived from luminal lactose as a measure of metabolic competence were recorded every 15 minutes; measurements were carried out by standard photometric assays. The lactate-to-pyruvate ratio as a measure of aerobic metabolism was determined in the venous outflow as described before [3].

### Analysis of peristalsis

The intestines were filmed by a standard digital miniature camera for offline video analysis. Overall motility in all sections of the intestines, before stimulation with PAF and at the end of the experiment, was evaluated. The time point of first segmental appearance of atony and complete atony as well as the restart of peristalsis were noted.

### Chemicals and perfusates

Perfusates were mixed from stock solutions and solid components on a daily basis and were pH adjusted and sterile filtered. For the composition of perfusates, see S2 Table. All chemicals were obtained from Sigma-Aldrich (Munich, Germany) if not otherwise stated.

### Histologic examination

Hematoxylin and eosin (HE)- and periodic acid-Schiff (PAS)-stained sections, for goblet cell mucus identification, were examined in a blinded fashion. The histological stability score was assessed as described in detail before [3]. Additionally, we studied the acute changes in morphology after PAF-stimulation with/without protective treatment in the PAS-stained sections. The differences in edema formation in the mucosa and the gut wall were analyzed 3 minutes after the challenge with PAF.

### Eicosanoid ELISA

Thromboxane and leukotrienes were analyzed in the venous effluent samples with a monoclonal Thromboxane B2 Express EIA kit and a Cysteinyl Leukotriene Express EIA kit (both Cayman Chemical, Ann Arbor, MI, USA) according to the manufacturer’s instructions.

### Parameters of the model stability

The histological stability score, the wet-to-dry weight and the lactate-to-pyruvate ratios at the end of experiment were calculated as described [3]. These data are presented in the S4 Fig., S5 Fig. and S6 Fig. and document stable conditions in all experiments. In all the groups the histological stability score, the wet-to-dry weight and the lactate-to-pyruvate-ratios at the end of the experiments were in a physiological range and comparable.

### Statistics

All data are given as means ± SD, except for the pressure responses and the duration of intestinal atony which are plotted as means ± SEM. Prior to statistical analysis using Prism (GraphPad Prism version 5.01 for Windows, GraphPad Software, San Diego California, USA), data were transformed to achieve homoscedasticity, if applicable.

Pressure responses were compared by calculating the area under the curve (AUC) of the respective pressure normalized to the pressure at 60 minutes within 15 minutes after the challenge with PAF. These data were transformed using the square root transformation to achieve homoscedasticity. The data of the FITC dextran distribution within the compartments were transformed using the arcsin transformation to achieve normality. One-way ANOVA with Tukey’s multiple comparison post test was used to calculate statistical significance of differences.

To calculate statistical significance of differences of the galactose uptake at time point 75 minutes and further, the parameters of the stability of the model at the end of the experiments, the Kruskal-Wallis test with Dunn’s multiple comparison test was used.

The changes of the wet-to-dry weight ratios within the PAF and the control groups acquired from the experiments with the duration of 63 minutes were analyzed by paired t-test. The histological stability scores at the end of these experiments were compared with unpaired t-test.

## Results

### Effects of Platelet-activating factor (PAF)

**Fig 1:**
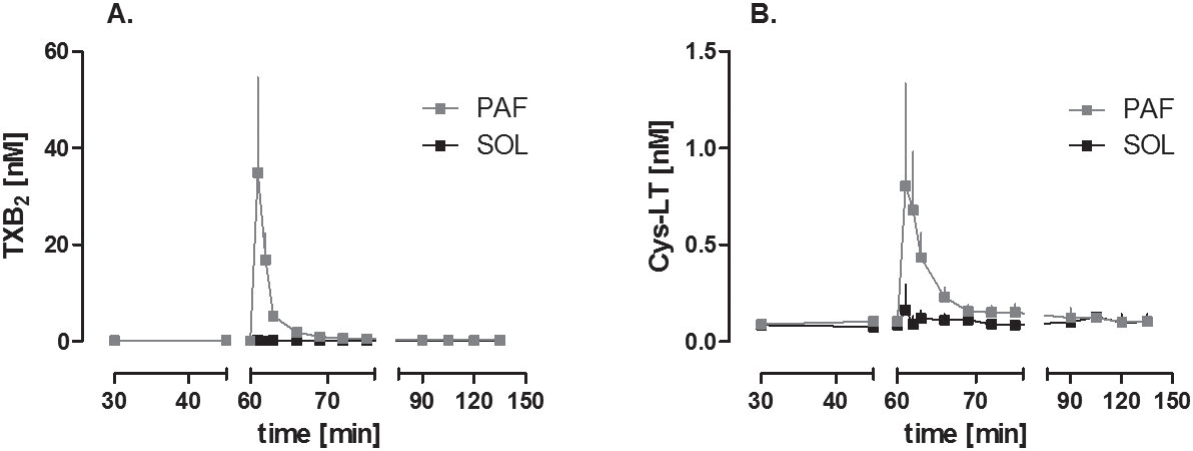
PAF-induced eicosanoid liberation to the vascular compartment. **A.** Concentration of thromboxane in the venous compartment of PAF-stimulated intestines (PAF, n = 3) and untreated intestines (SOL, n = 4); **B.** Concentration of cysteinyl leukotrienes in the venous compartment of PAF-stimulated intestines (PAF, n = 3) and untreated intestines (SOL, n = 4).

**Fig 2:**
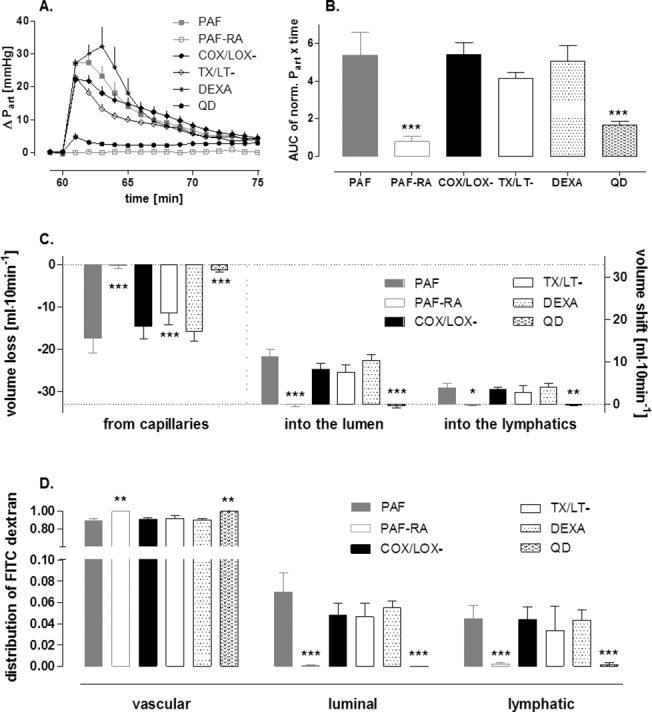
Intestinal physiology: PAF-induced vasoconstriction and permeability—Effects of eicosanoid antagonists, dexamethasone and quinidine. Vascular pressure tracings, intestinal fluid and macromolecule distributions in response to platelet-activating factor alone (PAF, n = 5) or in parallel with pretreatment by a PAF receptor antagonist (PAF-RA, n = 4), COX and LOX inhibitors (COX/LOX-, n = 5), thromboxane and leukotriene receptor antagonists (TX/LT-, n = 6), dexamethasone (DEXA, n = 6) or by quinidine (QD, n = 8). **A.** Vascular pressure response; **B.** Area under the pressure response curve, for calculations see Methods; **C.** Fluid distribution in the isolated intestine, columns at the left side: loss of fluid from vascular circulation, in the middle: volume shift into the lumen, columns at the right side: volume shift into the lymphatics; **D.** Distribution of FITC-labeled dextran in the isolated intestine (fractions). One-way ANOVA with Tukey’s multiple comparison post test was used to calculate statistical differences (* p<0.05 versus PAF; ** p<0.01 versus PAF; *** p<0.0001 versus PAF).

**Fig 3:**
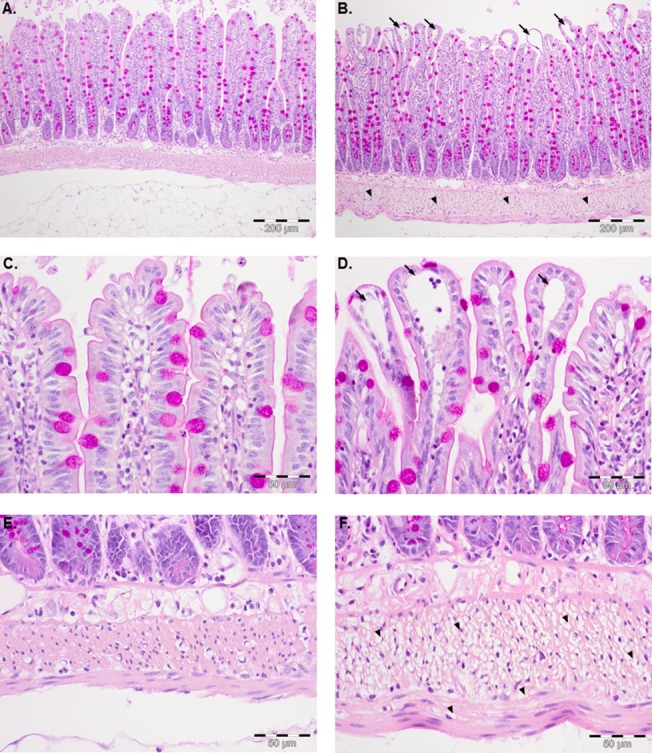
Histomorphology of normal intestines and of PAF-induced intestinal edema. Periodic acid-Schiff (PAS)-stained sections of longitudinal intestinal slices. **A., C. and E.** Normal morphology in control specimens at 63 minutes (n = 4). **B., D. and F.** The slide shows the acute morphologic changes in the intestinal mucosa and the gut wall three minutes after stimulation with PAF (n = 4). Fluid accumulations at the villi tips (arrows) and in the muscular layers (arrowheads) can be observed.

**Fig 4:**
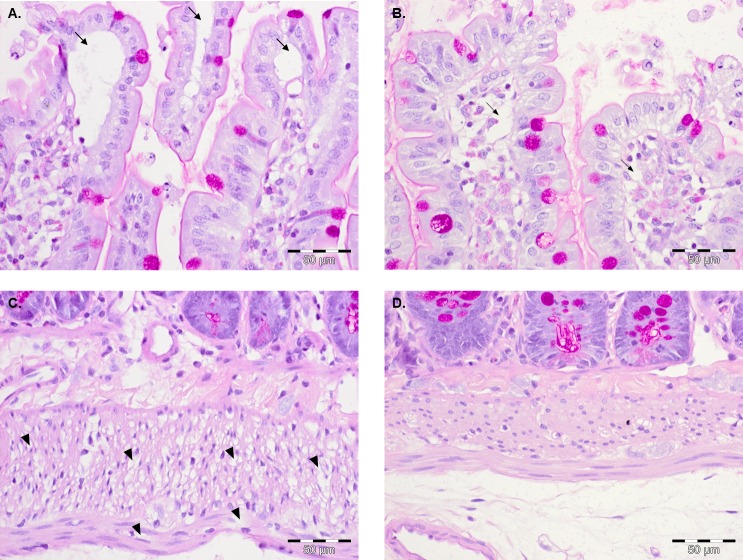
Histomorphology of PAF stimulated intestines pretreated with dexamethasone or quinidine. PAS-stained sections of longitudinal intestinal slices. **A.** Fluid accumulations at the villus tips (arrows) of PAF-stimulated intestines after pretreatment with dexamethasone (n = 1); **B.** Slight fluid accumulations at the villus tips (slim arrows) of PAF-stimulated intestines after pretreatment with quinidine (n = 1); **C.** Fluid accumulations in the intestinal muscular wall (arrowheads) of PAF-stimulated intestines after pretreatment with dexamethasone (n = 1). **D.** Normal morphology without fluid accumulations in the intestinal muscular wall of PAF-stimulated intestines after pretreatment with quinidine (n = 1).

**Fig 5:**
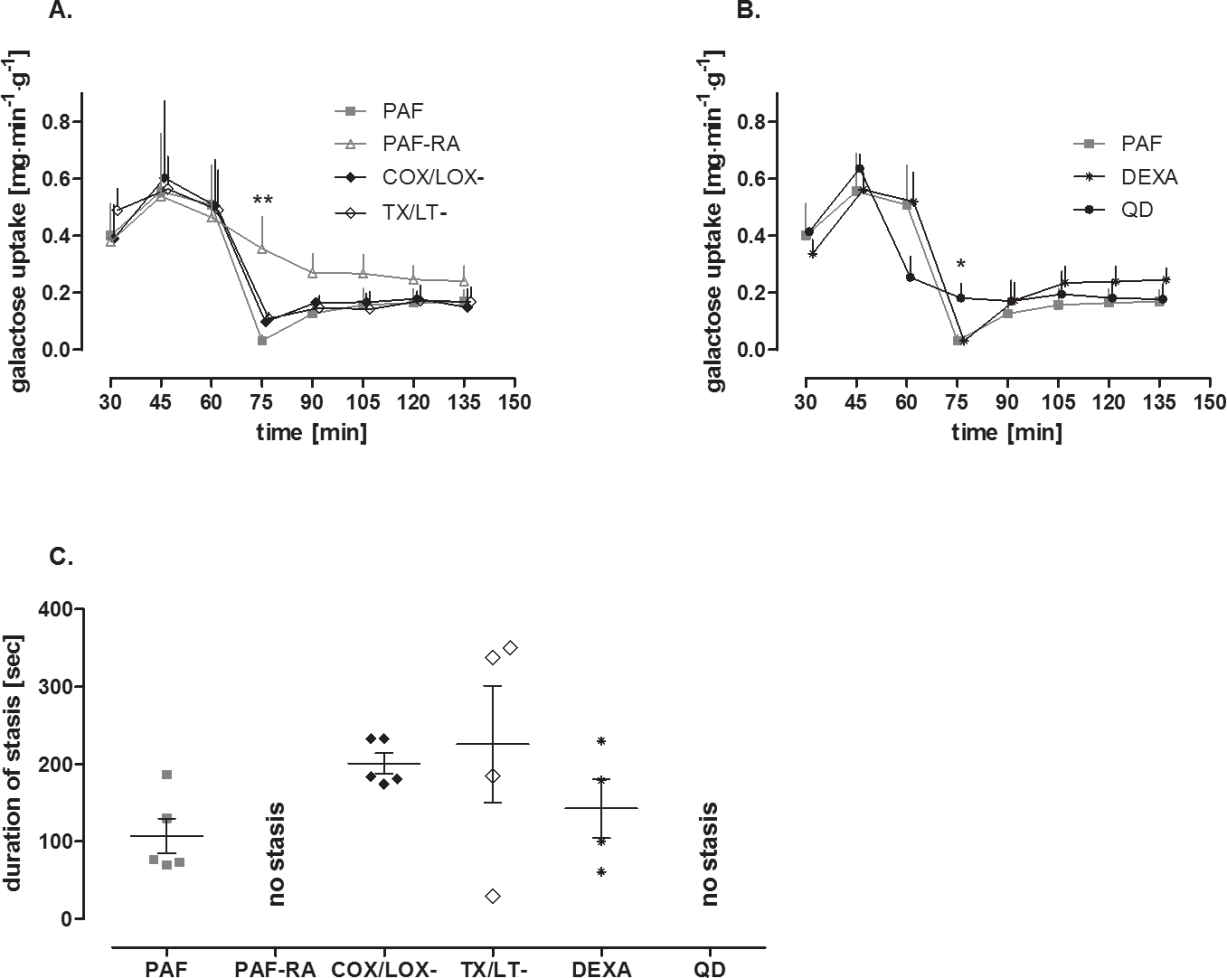
Intestinal functions: PAF-induced loss of digestive function and intestinal paralysis—Effects of eicosanoid antagonists, dexamethasone and quinidine. Tracings of galactose uptake and analysis of intestinal motility in response to PAF alone (PAF, n = 5) or in parallel with treatment by a PAF receptor antagonist (PAF-RA, n = 4), COX and LOX inhibitors (COX/LOX-, n = 5), thromboxane and leukotriene antagonists (TX/LT-, n = 6), dexamethasone (DEXA, n = 6) or by quinidine (QD, n = 8). **A. and B.** Tracings of galactose uptake in the portal vein derived from luminal lactose. At t = 75minutes Kruskal-Wallis test, with Dunn’s multiple comparison test was performed and all treatments were compared with PAF. PAF-RA ** and QD * differed significantly (* p<0.05 versus PAF; ** p<0.01 versus PAF); **C.** Analysis of paralysis. Duration of stasis is plotted as mean+SEM of each group when analysis was possible. In two experiments of the DEXA group, in one experiment of the PAF-RA group and in one experiments of the TX/LT- group no videos could be taken due to technical problems. In the PAF-RA and the QD group peristalsis was preserved (no stasis). In a single experiment of the TX/LT- group peristalsis was reduced but no stasis was seen.

Administration of a 0.5 nmol PAF bolus into the mesenteric artery liberated thromboxane and leukotriene from the intestine into the vascular compartment (Fig. 1). It caused a rapid and strong vasoconstriction (Fig. 2A and 2B) and a remarkable shift of fluid with a dramatic loss of vascular volume equivalent to about 13% of the total vascular fluid flow (Fig. 2C). In addition there was a transfer of FITC-labeled dextran from the vascular compartment to the lumen and the lymphatics (Fig. 2D), behaving similar as FITC-labeled albumin [3]. In the experiments in which the acute effects of PAF were analyzed, we observed intestinal edema located at the villus tips (Fig. 3B, 3D and Fig. 4A) and in the circular and longitudinal muscular layers (Fig. 3F and Fig. 4C). In these experiments, edema formation was also documented by a significant increase in the wet-to-dry weight ratio in the PAF group (4.02±0.19 to 4.79±0.17, p<0.05; paired t-test; n = 4), while, in the control group, there was just a slight change in the wet-to-dry weight ratio (4.05±0.26 to 4.31±0.24, p>0.05; paired t-test; n = 4). Of note, the histological stability scores of the intestines were not altered three minutes after the administration of PAF in comparison to controls (0.94±0.02 vs 0.97±0.01, p>0.05; unpaired t-test; n = 4), implying that the rapid effects of PAF depend on specific effects rather than on tissue damage. In addition, intestinal function, as monitored by the uptake of galactose derived from luminal lactose to the portal vein, was impaired within 15 minutes after the PAF administration and recovered thereafter (Fig. 5A). PAF reduced peristalsis within seconds (33±6 seconds) up to a period of complete atony (after 49±4 seconds, duration 97±50 seconds, Fig. 5C) with total recovery.

All these changes in microcirculatory and intestinal functions as well as morphological alterations were entirely reversible within the observation period of 135 minutes. All effects of PAF were abolished by the PAF receptor antagonist ABT 491 (Fig. 2A-D, Fig. 5A and 5C).

### Effects of eicosanoid antagonism on PAF-induced intestinal disorders

PAF increased the concentrations of thromboxane and leukotriene in the vascular compartment (Fig. 1). However, neither cyclooxygenase or lipoxygenase inhibitors nor thromboxane or leukotriene receptor antagonists affected the PAF-induced vasoconstriction (Fig. 2A and 2B). There was a small—and in the case of the receptor antagonists—significant effect on the PAF-induced volume loss from the capillaries (Fig. 2C) while the transfer of FITC-labeled dextran into the lumen (Fig. 2D) and the PAF-induced impairment of galactose uptake were not improved (Fig. 5A). The dynamics of the reduction of peristalsis was similar to PAF alone (group COX/LOX-: reduction of peristalsis after 31±6 seconds, atony after 44±4 seconds; group LT/TX-: reduction of peristalsis after 35±4 seconds, atony after 54±19 seconds), but the duration of atony was doubled (Fig. 5C; group COX/LOX-: atony for 201±29 seconds; group LT/TX-: atony for 225±151 seconds). Of note, in a single experiment in the group TX/LT- no atony was seen.

### Effects of dexamethasone on PAF-induced intestinal disorders

Dexamethasone treatment had no effect on the PAF-induced vasoconstriction, fluid shifts and transfer of FITC-labeled dextran (Fig. 2A-D). Dexamethasone also failed to prevent edema formation (Fig. 4A and 4C). It did not improve the PAF-induced loss of the intestinal digestive function (Fig. 5B) and it did not ameliorate the reduction in peristalsis which started after 30±7 seconds, was complete after 50±5 seconds and lasted for 142±76 seconds (Fig. 5C). This was even longer than in the absence of the steroid. Similar to controls, all effects of PAF were reversible in the presence of dexamethasone and at the end of the experiments the histological stability score exhibited normal values of 0.95±0.05 and the wet/dry ratio was 4.39±0.32 in the dexamethasone group.

### Effects of quinidine on PAF-induced intestinal disorders

The PAF-induced vasoconstriction, the fluid shift and the transfer of FITC-labeled dextran were nearly abolished in the quinidine group (Fig. 2A-D). Quinidine-treated specimens exhibited different histological findings in the mucosa and the intestinal wall. The intestinal villi appeared less edematous than without quinidine (Fig. 4B vs Fig. 3D), but they were not as slim as in controls (Fig. 3C), while the muscular layers looked normal (Fig. 4D) as in controls (Fig. 3E). The PAF-induced loss of galactose uptake was abolished by quinidine (Fig. 5B) and atony was prevented (Fig. 5C). Of note, we found a decrease in galactose uptake after the initiation of quinidine pretreatment even before PAF stimulation (Fig. 5B, value at time point 60 minutes). The PAF-induced reduction of peristalsis was delayed in one half of the experiments (48±14 s) and totally abolished in the rest. In none of the experiments with quinidine did we find complete atony (Fig. 5C). Total recovery of peristalsis had occurred at the end of all experiments and the histological stability score obtained at the end of the experiments was normal (0.93±0.1).

## Discussion

Intestinal disorders are frequently characterized by vasoconstriction, barrier disruption and (paralytic) ileus—symptoms that are reproduced by administration of platelet activating-factor (PAF) in the isolated intestine [3]. Therefore, we believe that this model is useful to evaluate the effectiveness of anti-inflammatory therapies and to develop drugs for a variety of intestinal ailments.

### PAF-induced vasoconstriction

The PAF-induced increase in mesenteric vascular resistance has been linked to bowel necrosis [9,31]. In our model of the flow-constant perfused small intestine, vascular resistance is a measure of the sum of arterial, capillary and venular diameter. The method does not allow to distinguish between pre- and post-capillary response, but a number of observations indicate that PAF most likely acts on mesenteric veins, including: (i) PAF increases intracellular calcium and tension in only mesenteric venous smooth muscle cells [32]; (ii) PAF fails to constrict isolated mesenteric arteries [33]; and (iii) topical application of PAF to the serosa or mucosa induces post-capillary vasoconstriction *in vivo* [5].

In one previous study the PAF-induced mesenteric vasoconstriction was reduced by treatment with FPL 55712 [34], a mixed leukotriene receptor antagonist and phosphodiesterase (PDE) inhibitor [35]. While the present data confirm that PAF can release leukotrienes, our experiments using specific antagonists clearly show that leukotrienes or thromboxane play no role in the PAF-induced contraction of the mesenteric vessels. Therefore, it appears likely that the effects of FPL 55712 are explained by the increase in cAMP or cGMP levels due to PDE inhibition [34].

The remarkable finding that quinidine almost completely prevented the PAF-induced vasoconstriction—similar to the drug’s effects in the lungs [18]—is at present difficult to interpret in mechanistic terms. Quinolines such as quinidine and hydroxychloroquine are long known for their anti-inflammatory properties and there is growing evidence for their beneficial effects in various diseases, among them metabolic and cardiovascular disorders [36]. Unfortunately, their mechanism of action remains poorly defined; possible modes of action relate to the inhibition of ion channels [37] or IP3-dependent calcium signaling [38].

In consideration of the present findings and previous published studies we propose the following hypothesis: In vascular beds where PAF contracts the arteries such as in the lungs and in the hamster cheek pouch, PAF-induced vasoconstriction is mediated by thromboxane and leukotrienes [39,40]. In organs where only veins contract, as most likely in the intestine [32,33,41] (mesenteric arteries, may even relax [42]), eicosanoids do not play a significant role. In such organs, the vasoconstriction is possibly mediated by the direct activation of PAF receptors on venous smooth muscle cells.

### PAF-induced edema formation

PAF is one of the few mediators that can raise the endothelial permeability within minutes [43,44]. The increase in vascular permeability [3,45] leads to edema formation, as was confirmed here by the weight recordings, the transfer of FITC dextran to the lymphatics and the lumen, and by the histomorphologic alterations. In addition to increasing vascular permeability, PAF causes dilation of mesenteric lymph vessels and decreases their contraction frequency [46], thereby limiting the lymph transport and aggravating the swelling. Swelling of the intestine is a serious clinical problem and may cause suture dehiscence and abdominal compartment syndrome. Therefore, drugs preventing the intestinal swelling would be highly useful.

Steroids are widely considered as potent anti-edematic agents. For example, they prevent PAF-induced edema formation in the isolated lungs [19,47], the hamster cheek pouch [48], the rabbit skin [49] and they also prevent edema formation provoked by other stimuli like capsaicin or arachidonic acid [50]. Therefore, the inability of dexamethasone to protect against the PAF-induced edema was unexpected. However, to our knowledge, the effect of steroids on PAF-induced intestinal edema have not been studied before. There are only two related studies showing that dexamethasone reduced the macroscopically apparent gastrointestinal damage following i.v. PAF injection [51,52]. However, both studies did not allow conclusions on edema formation and the protection by dexamethasone is likely explained by its effects on leukocyte recruitment [52]. Interestingly, a similar lack of barrier protection by steroids as described here has been reported in the hindpaw [53]. These findings may be part of the explanation why steroids are of little help in sepsis and other disorders with intestinal edema.

Our conclusions regarding the role of leukotrienes are similar to that of steroids. There exists evidence that anti-leukotriene strategies may be effective in preventing intestinal damage [13,15]; however, this has not yet been demonstrated for PAF-induced intestinal edema. Similar to steroids, in the present study leukotriene antagonists were ineffective in the intestine. In the lungs, we have shown that PAF-induced edema formation is in part mediated by COX-1 derived [54] prostaglandin E_2_ acting on EP3 receptors [27]. Our present findings rule out any role of PGE_2_, leukotrienes or of thromboxane as mediators of the PAF-induced edema in the intestine. Of note, high doses of the COX-inhibitor indomethacin may even increase intestinal permeability [7].

In contrast to steroids and drugs interfering with eicosanoids, quinidine was highly effective in preventing the PAF-induced edema in the intestine, similar to its effects in the lungs [18]. The mechanisms behind this protection are still unknown. It is remarkable that quinidine is able to prevent the PAF-induced edema formation in both the intestine and the lungs because the mechanisms of edema formation in these organs are rather different [44]; a difference which is also supported by the ineffectiveness of dexamethasone in the present study (Table 1). These findings suggest that quinidine and similar drugs should be considered for the treatment of systemic inflammatory diseases. In addition to the anti-edematous effects shown here, these agents also reduce the levels of pro-inflammatory cytokines [55,56] and possess anti-pyretic properties [57]. The therapeutic potential of quinolines in models of septic shock is supported by several studies showing protection in LPS-induced lung injury [18], LPS-induced death [58], galactosamine/endotoxin-induced hepatitis [28] and carrageenan-induced hindpaw inflammation [59].

**Table 1:** Effects of drugs on organ dysfunction in the isolated perfused rat intestine and in the isolated perfused rat lung.

	Vasoconstriction		Edema formation		Paralysis
	intestine	lung	intestine	lung	intestine
COX/LOX inhibition	NO	YES	NO	YES	NO
TX/LT antagonist	NO	YES	NO	NO	NO
Dexamethasone	NO	NO	NO	YES	NO
Quinidine	YES	YES	YES	YES	YES

Cyclooxygenase (COX); Lipoxygenase (LOX); Thromboxane (TX); Peptidoleukotrienes (LT); the data for the intestine are from this paper and the data for the lung are from the references [18,19,39].

### Paralysis

Impaired gastrointestinal motility and paralysis, also known as septic paralytic ileus, is a well-known complication of sepsis and SIRS [60]. It has been shown that LPS-induced intestinal motor disturbances are in part mediated by PAF [61,62]. However, the mechanisms of the PAF-induced cessation of peristalsis are almost completely unknown. Our findings show that this effect of PAF occurs independent of blood-borne leukocytes and eicosanoids, and cannot be prevented by steroids. Remarkably, however, this effect of PAF was largely blocked by quinidine, implying again that this drug may be useful to treat septic complications of the gastrointestinal tract.

### Quinidine

The cinchona alkaloids quinine, quinidine, cinchonine and cinchonidine are traditionally used to treat different inflammatory diseases [63]. Here we show that quinidine counteracts all of the PAF-induced dysfunctions observed in this study: mesenteric vasoconstriction, capillary leak, intestinal edema, cessation of peristalsis and loss of galactose uptake. At present, the molecular basis for these beneficial effects is unknown. Multiple targets for quinolines have been identified such as ion channels [37] or IP-receptors [38], but a consistent picture to explain its beneficial properties is not yet apparent.

Regardless of the mechanisms, quinolines may be useful in sepsis and in inflammatory bowel disease [64,65]. These drugs appear attractive because they are broadly anti-inflammatory, effective in many animal models and unlike steroids they also prevent the PAF-induced dysfunctions in the intestine. Notwithstanding the fact that quinolines have a small therapeutic index and may evoke central nervous, cardiovascular, gastrointestinal and metabolic side effects [23], they are currently used for the treatment of acute malaria, rheumatoid arthritis, systemic lupus erythematosus and various rheumatic diseases [36]. Moreover, quinidine as an antiarrhytmic drug has still clinical relevance in the treatment of low-prevalence diseases like Brugada syndrome, idiopathic ventricular fibrillation, early repolarization syndrome and short-QT syndrome [66]. Our study strongly suggests that further investigations are warranted into the clinical role of quinolines in inflammatory disorders, especially in SIRS and sepsis.

### Conclusion

The present study closes an important gap in our knowledge in that it shows that neither steroids nor anti-leukotriene strategies prevent any of the PAF-induced dysfunctions in the intestine. Our study also illustrates that PAF—despite causing vasoconstriction and edema formation with nearly identical kinetics—employs distinct mechanisms in the intestine and the lungs. These findings have immediate clinical implications because they indicate how difficult it will be to treat systemic inflammatory diseases such as sepsis with a single drug. Notably, quinidine was the only drug effective in the intestine and in the lungs, suggesting that quinolines may be therapeutically useful in diseases with both intestinal and pulmonary dysfunctions.

## Supporting Information

S1 Fig Experimental rational and potential inflammatory signal transduction of PAF. It is commonly thought that the signal transduction of PAF is mediated by secondary eicosanoids. Intestinal thromboxane and leukotrienes may also lead to vasoconstriction, permeability and edema as well as paralysis and organ failure. As a first step, PAF-induced intestinal eicosanoid delivery and pathophysiological consequences were measured. Secondly, blockade of each potential step in signal transduction and more general anti-inflammatory acting agents were used to clarify the relevance in the intestine. ABT 491, PAF-receptor antagonist. ASA, acetylsalicylic acid (aspirin), inhibitor of cyclooxygenase. AA 861, inhibitor of lipoxygenase. SQ 29548, thromboxane-receptor antagonist. MK 571, leukotriene-receptor antagonist. ELISA, measurement of PAF-induced intestinal eicosanoid delivery by enzyme linked immunosorbent assay. Dexamethasone, corticoid-receptor agonist. Quinidine, chinoline derivate, anti-inflammatory treatment.
(TIF)

S2 Fig Experimental protocol. Bold vertical dashes represent time points when samples were taken for measurements of intestinal physiology. Regular vertical dashes represent time points when samples were taken for measurements of FITC dextrane transfer and eicosanoid production. continuously (cont.); minute (´)
(TIF)

S3 Fig Schematic drawing of the perfusion system. All intestinal compartments are accessible for detailed analysis. The pressure, the flow rate and the transfer of fluid, sugar or macromolecules can be detected. Fluorescein isothiocyanate (FITC).
(TIF)

S4 Fig Histological stability score. After 140 minutes of isolated perfusion the histological stability scores were calculated for all experiments. Intestines were stimulated with PAF alone (PAF, n = 5) or after pretreatment with a PAF receptor antagonist (PAF-RA, n = 4), without any stimulation or treatment (SOL, n = 5), after pretreatment with COX and LOX inhibitors (COX/LOX-, n = 5), thromboxane and leukotriene receptor antagonists (TX/LT-, n = 6), dexamethasone (DEXA, n = 6) or quinidine (QD, n = 8). Statistics were calculated with Kruskal-Wallis test and Dunn’s multiple comparison test; no significant differences versus PAF.
(TIF)

S5 Fig Wet-to-dry weight ratio. After 140 minutes of isolated perfusion the wet-to-dry weight ratios were calculated for all experiments. Intestines were stimulated with PAF alone (PAF, n = 5) or after pretreatment with a PAF receptor antagonist (PAF-RA, n = 4), without any stimulation or treatment (SOL, n = 5), after pretreatment with COX and LOX inhibitors (COX/LOX-, n = 5), thromboxane and leukotriene receptor antagonists (TX/LT-, n = 6), dexamethasone (DEXA, n = 6) or quinidine (QD, n = 8). Statistics were calculated with Kruskal-Wallis test and Dunn’s multiple comparison test; no significant differences versus PAF.
(TIF)

S6 Fig Lactate-to-pyruvate ratio. After 140 minutes of isolated perfusion the lactate-to-pyruvate ratios were calculated for most experiments. Intestines were stimulated with PAF alone (PAF) or after pretreatment with a PAF receptor antagonist (PAF-RA), without any stimulation or treatment (SOL), after pretreatment with COX and LOX inhibitors (COX/LOX-), thromboxane and leukotriene receptor antagonists (TX/LT-), dexamethasone (DEXA) or quinidine (QD). Statistics were calculated with Kruskal-Wallis test and Dunn’s multiple comparison test; * p<0.05 versus PAF.
(TIF)

S1 Table Characteristics of the experimental groups. (DOC)

S2 Table Composition of perfusates. (DOC)
