# Supplementary figures and images for: Quinidine, but Not Eicosanoid Antagonists or Dexamethasone, Protect the Gut from Platelet Activating Factor-Induced Vasoconstriction, Edema and Paralysis
Source: PLoS One. 2015 Mar 20;10(3):e0120802. doi: 10.1371/journal.pone.0120802 (PMC4368623; doi:10.1371/journal.pone.0120802)

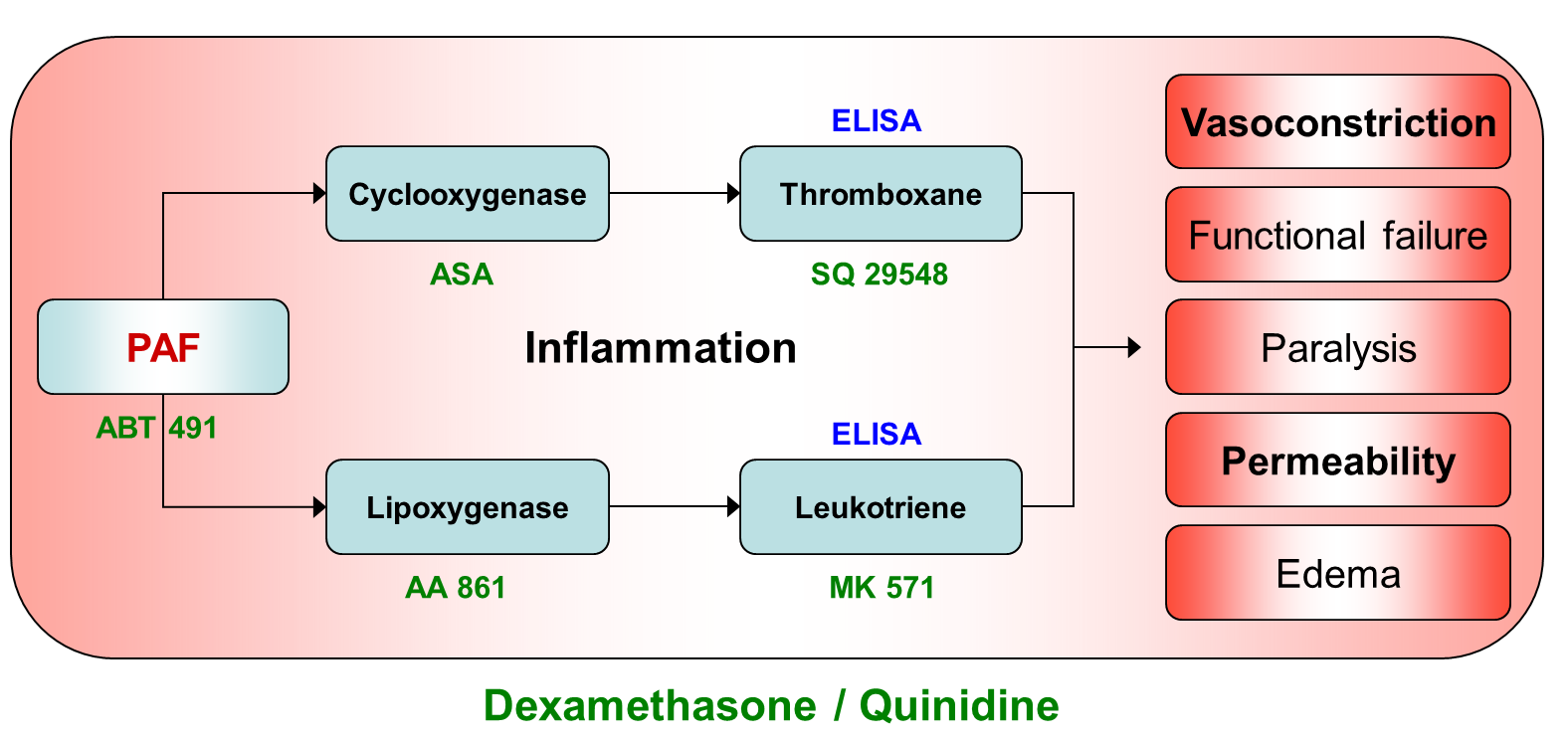

Supplement: S1 Fig — It is commonly thought that the signal transduction of PAF is mediated by secondary eicosanoids. Intestinal thromboxane and leukotrienes may also lead to vasoconstriction, permeability and edema as well as paralysis and organ failure. As a first step, PAF-induced intestinal eicosanoid delivery and pathophysiological consequences were measured. Secondly, blockade of each potential step in signal transduction and more general anti-inflammatory acting agents were used to clarify the relevance in the intestine. ABT 491, PAF-receptor antagonist. ASA, acetylsalicylic acid (aspirin), inhibitor of cyclooxygenase. AA 861, inhibitor of lipoxygenase. SQ 29548, thromboxane-receptor antagonist. MK 571, leukotriene-receptor antagonist. ELISA, measurement of PAF-induced intestinal eicosanoid delivery by enzyme linked immunosorbent assay. Dexamethasone, corticoid-receptor agonist. Quinidine, chinoline derivate, anti-inflammatory treatment. (TIF) [file pone.0120802.s001.tif]

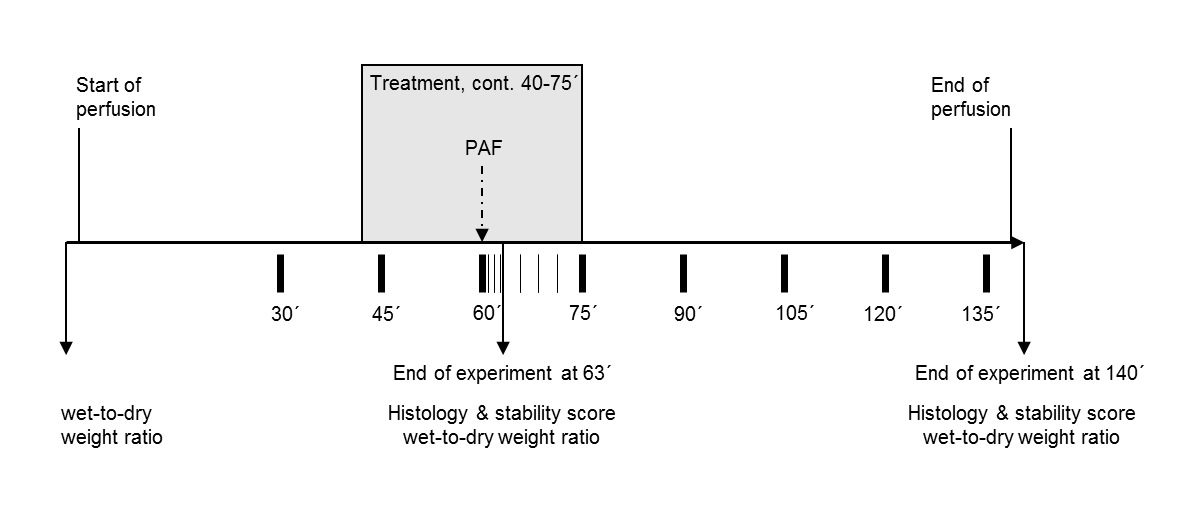

Supplement: S2 Fig — Bold vertical dashes represent time points when samples were taken for measurements of intestinal physiology. Regular vertical dashes represent time points when samples were taken for measurements of FITC dextrane transfer and eicosanoid production. continuously (cont.); minute (´) (TIF) [file pone.0120802.s002.tif]

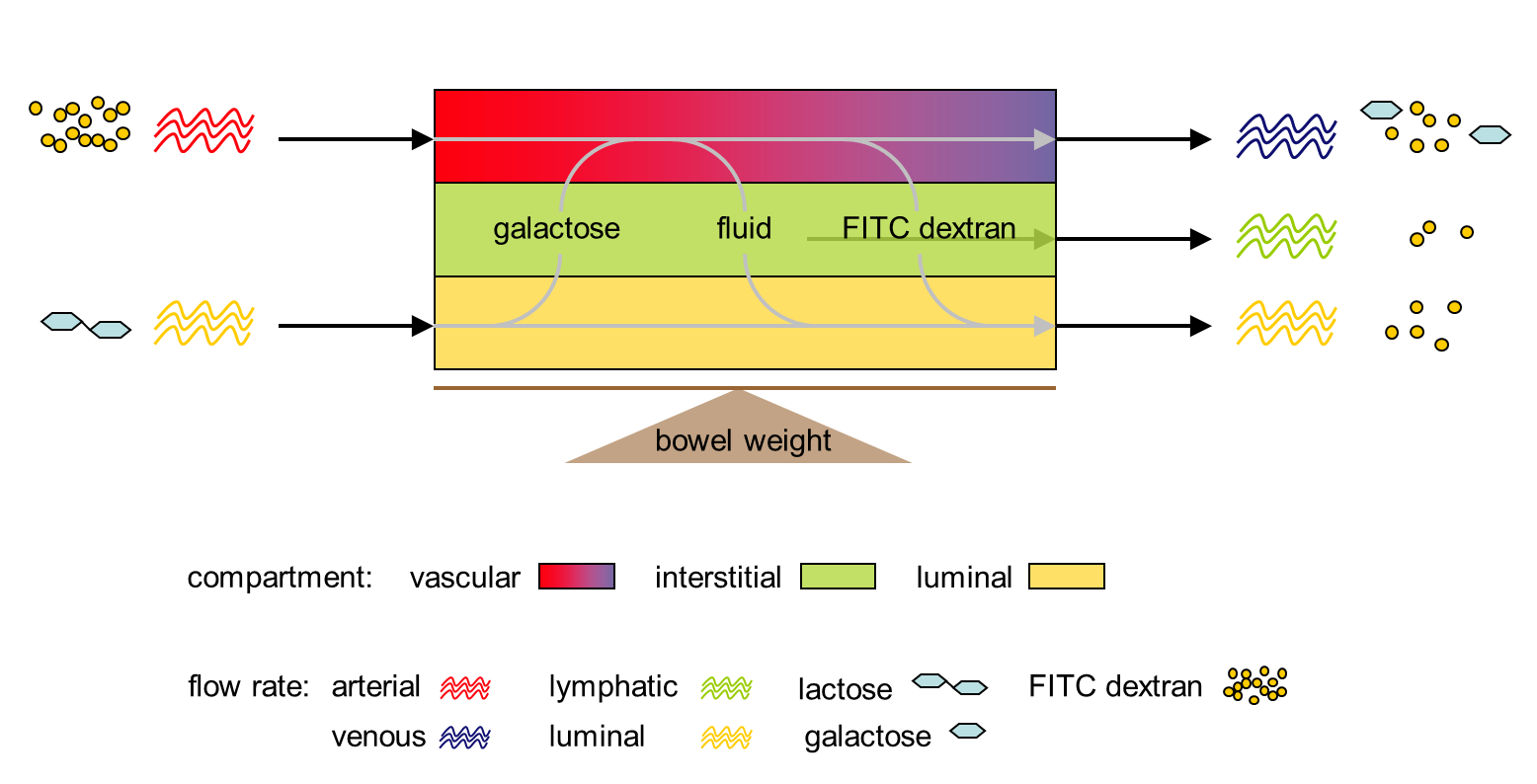

Supplement: S3 Fig — All intestinal compartments are accessible for detailed analysis. The pressure, the flow rate and the transfer of fluid, sugar or macromolecules can be detected. Fluorescein isothiocyanate (FITC). (TIF) [file pone.0120802.s003.tif]

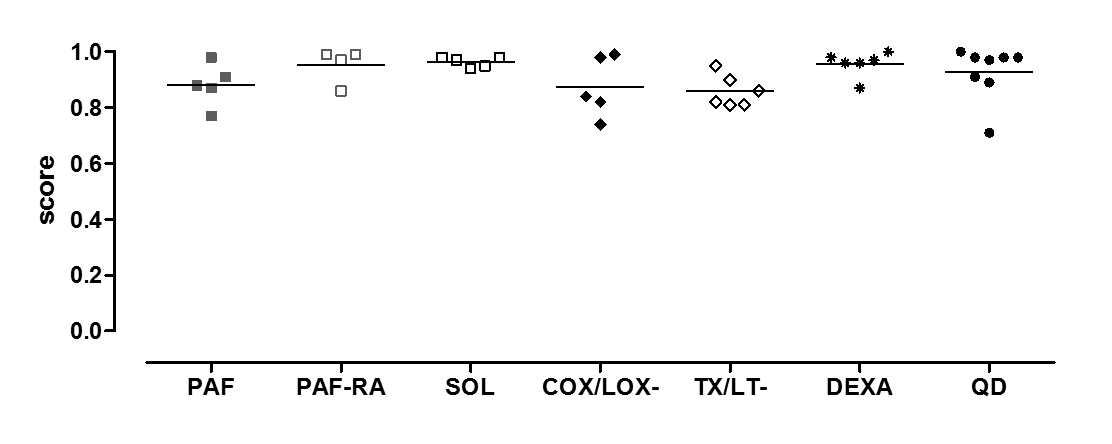

Supplement: S4 Fig — After 140 minutes of isolated perfusion the histological stability scores were calculated for all experiments. Intestines were stimulated with PAF alone (PAF, n = 5) or after pretreatment with a PAF receptor antagonist (PAF-RA, n = 4), without any stimulation or treatment (SOL, n = 5), after pretreatment with COX and LOX inhibitors (COX/LOX-, n = 5), thromboxane and leukotriene receptor antagonists (TX/LT-, n = 6), dexamethasone (DEXA, n = 6) or quinidine (QD, n = 8). Statistics were calculated with Kruskal-Wallis test and Dunn’s multiple comparison test; no significant differences versus PAF. (TIF) [file pone.0120802.s004.tif]

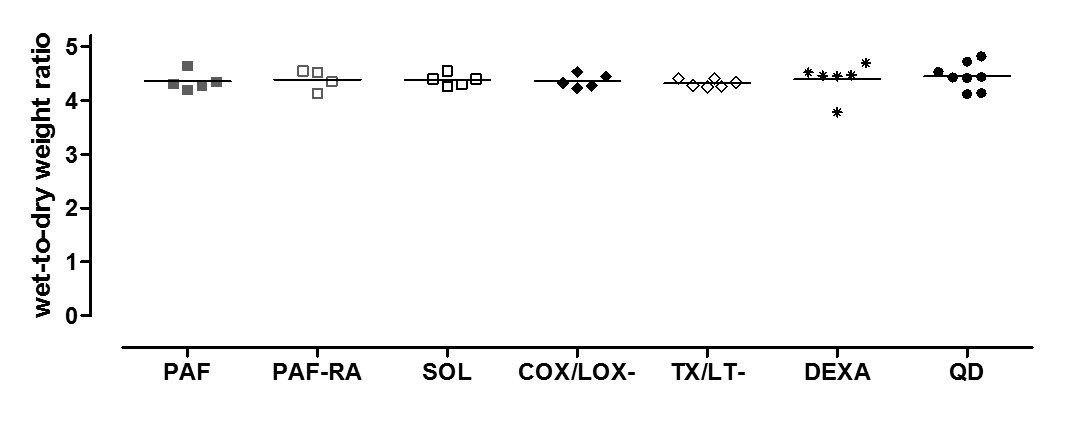

Supplement: S5 Fig — After 140 minutes of isolated perfusion the wet-to-dry weight ratios were calculated for all experiments. Intestines were stimulated with PAF alone (PAF, n = 5) or after pretreatment with a PAF receptor antagonist (PAF-RA, n = 4), without any stimulation or treatment (SOL, n = 5), after pretreatment with COX and LOX inhibitors (COX/LOX-, n = 5), thromboxane and leukotriene receptor antagonists (TX/LT-, n = 6), dexamethasone (DEXA, n = 6) or quinidine (QD, n = 8). Statistics were calculated with Kruskal-Wallis test and Dunn’s multiple comparison test; no significant differences versus PAF. (TIF) [file pone.0120802.s005.tif]

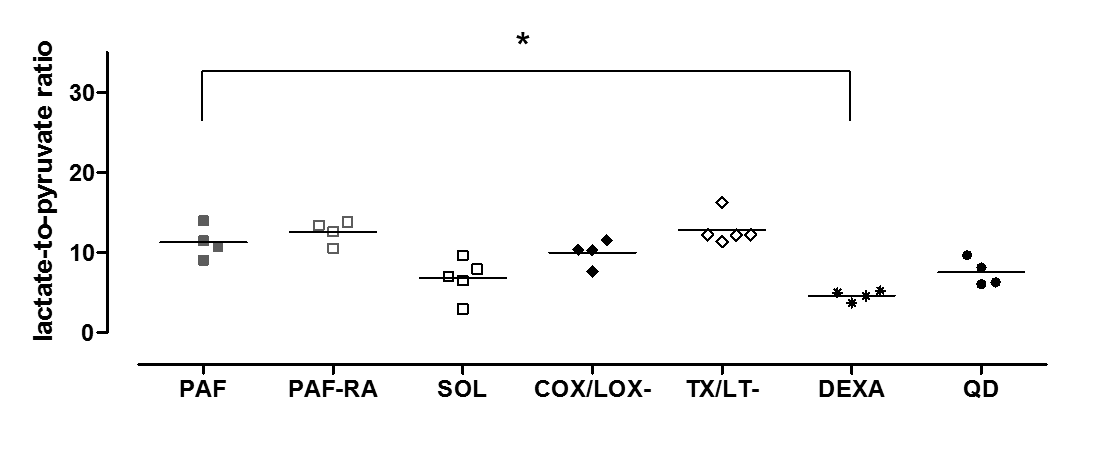

Supplement: S6 Fig — After 140 minutes of isolated perfusion the lactate-to-pyruvate ratios were calculated for most experiments. Intestines were stimulated with PAF alone (PAF) or after pretreatment with a PAF receptor antagonist (PAF-RA), without any stimulation or treatment (SOL), after pretreatment with COX and LOX inhibitors (COX/LOX-), thromboxane and leukotriene receptor antagonists (TX/LT-), dexamethasone (DEXA) or quinidine (QD). Statistics were calculated with Kruskal-Wallis test and Dunn’s multiple comparison test; * p<0.05 versus PAF. (TIF) [file pone.0120802.s006.tif]
