# Supplementary material for: Quinidine, but Not Eicosanoid Antagonists or Dexamethasone, Protect the Gut from Platelet Activating Factor-Induced Vasoconstriction, Edema and Paralysis
Source: PLoS One. 2015 Mar 20;10(3):e0120802. doi: 10.1371/journal.pone.0120802 (PMC4368623; doi:10.1371/journal.pone.0120802)
Supplement: S1 Table — (DOC) [file pone.0120802.s007.doc]

**Supporting Information Table S3. Characteristics** of the experimental groups.

| **Group (n)** | **Agent(s)** | **Amount or Concentration** | **Administration** | **Mechanism** |
| --- | --- | --- | --- | --- |
| ELISA-PAF (3) | PAF | 0.5 nmol | bolus | PAF-R |
| ELISA-SOL (4) | - | - | - | Control |
| PAF (5) | PAF | 0.5 nmol | bolus | PAF-R |
| PAF-RA (4) | ABT 491 | 2.5 µM | cont. 40-75´ | PAF-R |
| COX/LOX**-** (5) | ASA/AA861 | 500 µM/10 µM | cont. 40-75´ | Cyclooxygenase/ Lipoxygenase |
| TX/LT**-** (6) | SQ29548/MK571 | 10 µM/10 µM* | cont. 40-75´ | Thromboxane-R/ Leukotriene-R |
| DEXA (6) | Dexamethasone | 10 µM | cont. 40-75´ | Corticoid-R |
| QD (8) | Quinidine | 100 µM** | cont. 40-75´ | to be elucidated |
| SOL (5) | - | - | - | Control |
| *PAF-63 (4)* | *PAF* | *0.5 nmol* | *bolus* | *PAF-R* |
| *SOL-63 (4)* | *-* | *-* | *-* | *Control* |
| *DEXA-63 (1)* | *Dexamethasone* | *10 µM* | *cont. 40-63´* | *Corticoid-R* |
| *QD-63 (1)* | *Quinidine* | *100 µM* | *cont. 40-63´* | *to be elucidated* |

Replicates (n); receptor (R); continuously (cont.); minute (´); in one experiment concentration of agents were 30 µM/30 µM (*); in one experiment concentration of quinidine was 136 µM (**); *italic* = experiments with 63 minutes of perfusion for analysis of acute morphologic effects of PAF; in all experiments (except control) PAF was given as a bolus of 0.5 nmol at 60´.
