# Supplementary material for: Quinidine, but Not Eicosanoid Antagonists or Dexamethasone, Protect the Gut from Platelet Activating Factor-Induced Vasoconstriction, Edema and Paralysis
Source: PLoS One. 2015 Mar 20;10(3):e0120802. doi: 10.1371/journal.pone.0120802 (PMC4368623; doi:10.1371/journal.pone.0120802)
Supplement: S2 Table — (DOC) [file pone.0120802.s008.doc]

**Supporting Information Table S5**. Composition of perfusates.

|  | **Vascular perfusate** | **Luminal perfusate** |
| --- | --- | --- |
| Glucose, *mM* | 7.40 | 5.55 |
| Glutamine, *mM* | 0.80 | 0.80 |
| Lactose, *mM* | - | 30.00 |
| NaCl, *mM* | 95.00 | 114.00 |
| KCl, *mM* | 4.70 | 5.00 |
| KH_2_PO_4_, *mM* | 1.20 | - |
| MgSO_4_·7H_2_O, *mM* | 1.20 | - |
| CaCl_2_, *mM* | 2.50 | - |
| Lactobionic acid, *mM* | 2.00 | - |
| Mannitol, *mM* | 30.00 | 10.00 |
| HEPES,  *mM* | 12.60 | - |
| NaHCO_3_, *mM* | 25.00 | 26.00 |
| BSA, *mM* | 450.00 | - |
| pH (adjustment with 2M HCl/NaOH) | 7.52-7.58 | 7.50-7.58 |
| pH after equilibration with carbogen | 7.36±0.02 | - |
| Osmolarity, *mosmol/L* | 310-330 | 310-330 |
| L-Norepinephrine* *mM* | 5.91·10^-4^ | - |

* Sanofi-Aventis Deutschland GmbH, Frankfurt/Main, Germany.
